# Supplementary material for: Assessing the spatio-temporal risk of Aedes-borne arboviral diseases in non-endemic regions: The case of Northern Spain
Source: PLoS Negl Trop Dis. 2025 Jul 28;19(7):e0013325. doi: 10.1371/journal.pntd.0013325 (PMC12313078; doi:10.1371/journal.pntd.0013325)
Supplement: S3 Fig — (PDF) [file pntd.0013325.s003.pdf]

Assessing the Spatio-Temporal Risk of *Aedes*-Borne Arboviral Diseases in Non-Endemic Regions: The Case of Northern Spain

Bruno V. Guerrero *[a], Vanessa Steindorf[a], Rubén Blasco-Aguado[a], Luís Mateus[a], Aitor Cevidanes[b], Jesús F. Barandika[b], Ana Ramírez de La Peciña Pérez[c], Joseba Bidaurrazaga Van-Dierdonck[c], Jesús Angel Ocio Armentia[c], Nico Stollenwerk[a], and Maíra Aguiar †[a,d]

[a]BCAM - Basque Center for Applied Mathematics, Bilbao, Spain
[b]Animal Health Department, NEIKER-Basque Institute for Agricultural Research and Development, Basque Research and Technology Alliance (BRTA), Derio, Bizkaia, Spain
[c]Public Health, Basque Health Department, Rekalde Zumarkalea 39A, 48008 Bilbao, Spain
[d]Ikerbasque, Basque Foundation for Science, Bilbao, Spain

S3 Fig. Monthly maps of the expected number of imported viremic cases at the municipal level in the Basque Country for 2019, 2022, and 2023.

All maps were generated using shapefiles from the official resource provided by the Basque Government (Eusko Jaurlaritza / Gobierno Vasco), licensed under Creative Commons CC BY 4.0 https://www.euskadi.eus/limites-administrativos-del-pais-vasco/web01-ejeduki/es/.

*bguerrero@bcamath.org
†maguiar@bcamath.org

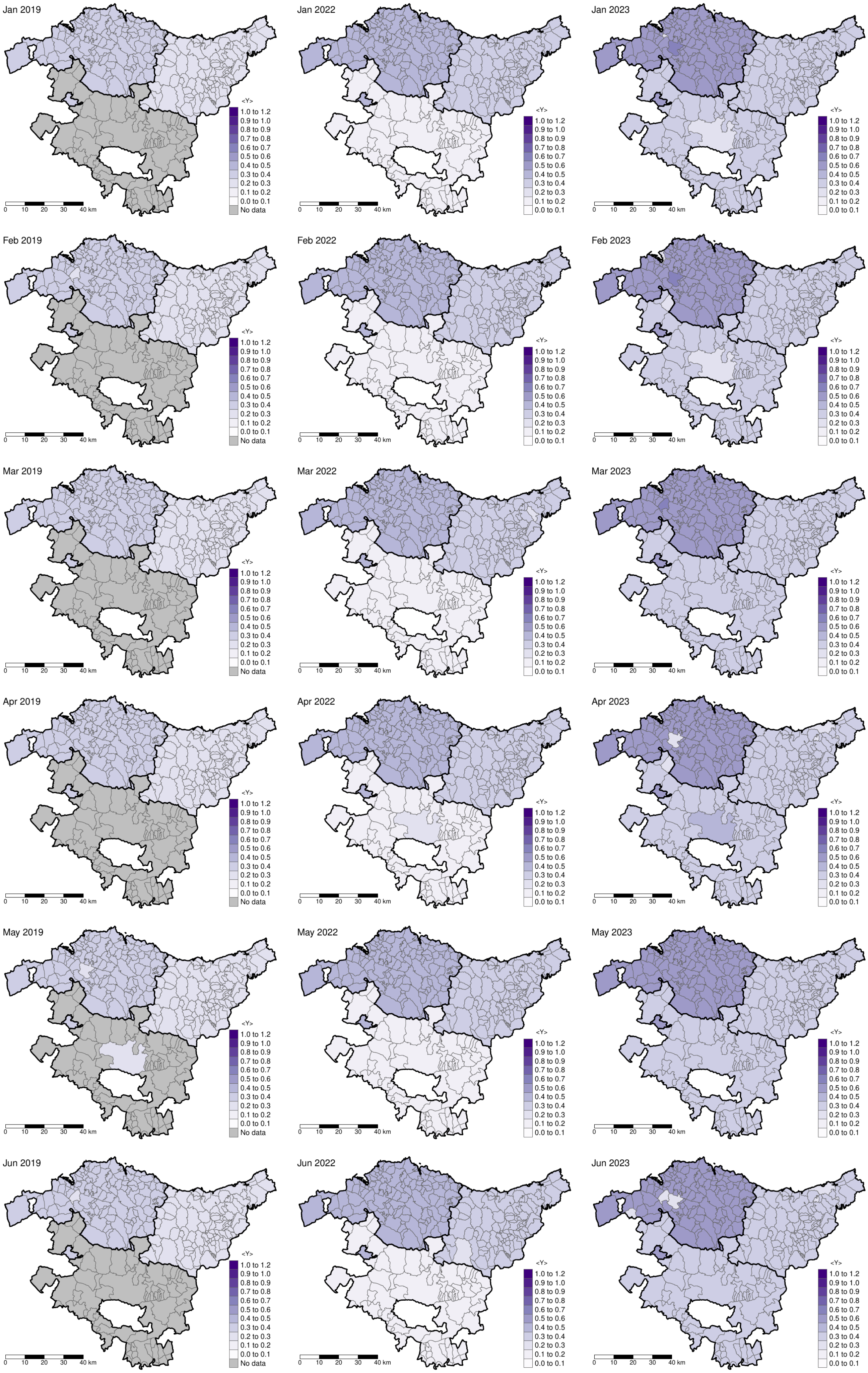
Jan 2019
Jan 2022
Jan 2023
Feb 2019
Feb 2022
Feb 2023
Mar 2019
Mar 2022
Mar 2023
Apr 2019
Apr 2022
Apr 2023
May 2019
May 2022
May 2023
Jun 2019
Jun 2022
Jun 2023
<Y>
1.0 to 1.2
0.9 to 1.0
0.8 to 0.9
0.7 to 0.8
0.6 to 0.7
0.5 to 0.6
0.4 to 0.5
0.3 to 0.4
0.2 to 0.3
0.1 to 0.2
0.0 to 0.1
No data
0
10
20
30
40 km

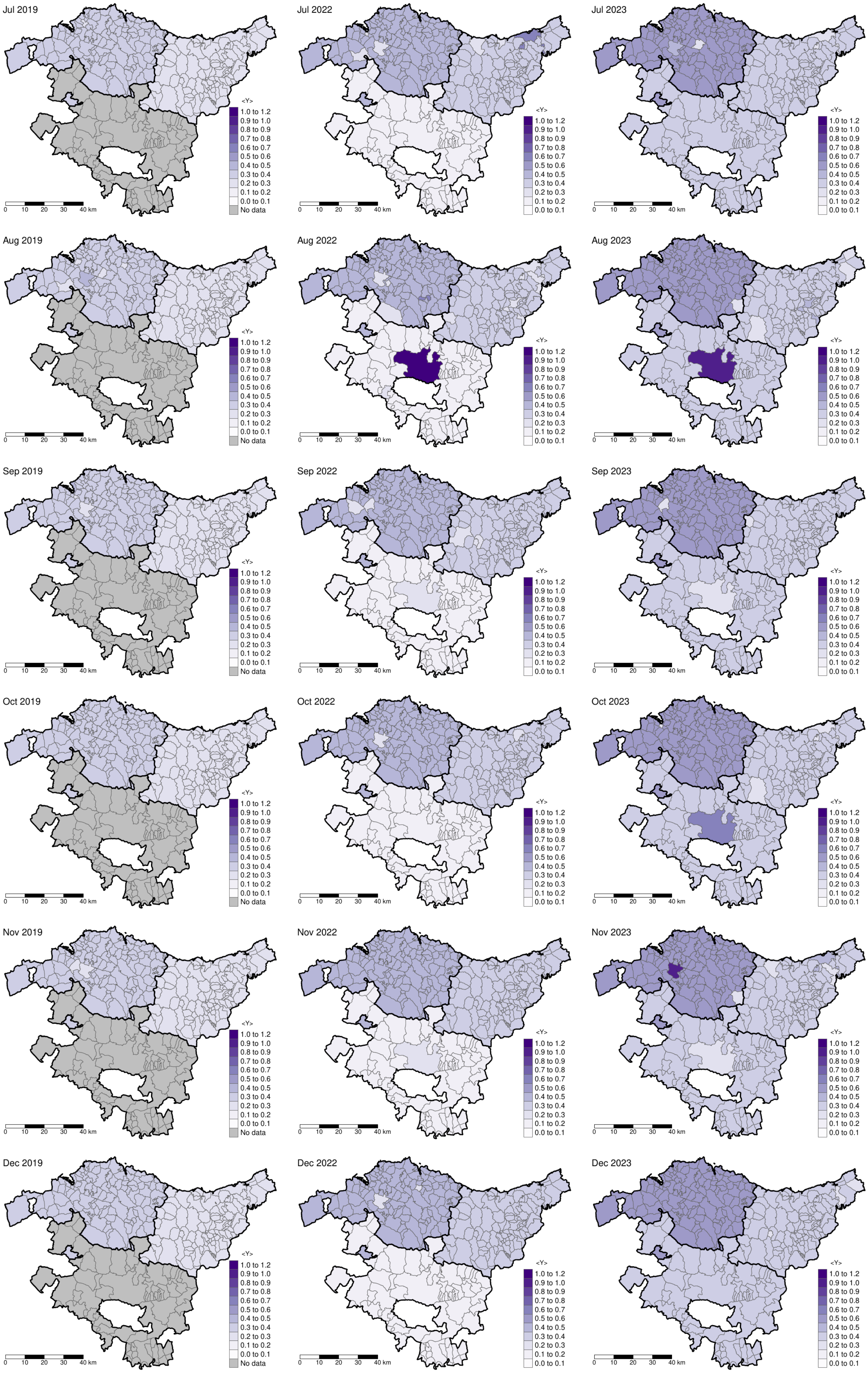
Jul 2019
Jul 2022
Jul 2023
Aug 2019
Aug 2022
Aug 2023
Sep 2019
Sep 2022
Sep 2023
Oct 2019
Oct 2022
Oct 2023
Nov 2019
Nov 2022
Nov 2023
Dec 2019
Dec 2022
Dec 2023
<Y>
1.0 to 1.2
0.9 to 1.0
0.8 to 0.9
0.7 to 0.8
0.6 to 0.7
0.5 to 0.6
0.4 to 0.5
0.3 to 0.4
0.2 to 0.3
0.1 to 0.2
0.0 to 0.1
No data
0 10 20 30 40 km
