# Supplementary material for: Assessing the spatio-temporal risk of Aedes-borne arboviral diseases in non-endemic regions: The case of Northern Spain
Source: PLoS Negl Trop Dis. 2025 Jul 28;19(7):e0013325. doi: 10.1371/journal.pntd.0013325 (PMC12313078; doi:10.1371/journal.pntd.0013325)
Supplement: S5 Fig — (PDF) [file pntd.0013325.s005.pdf]

Assessing the Spatio-Temporal Risk of *Aedes*-Borne Arboviral Diseases in Non-Endemic Regions: The Case of Northern Spain

Bruno V. Guerrero *[a], Vanessa Steindorf[a], Rubén Blasco-Aguado[a], Luís Mateus[a], Aitor Cevidanes[b], Jesús F. Barandika[b], Ana Ramírez de La Peciña Pérez[c], Joseba Bidaurrazaga Van-Dierdonck[c], Jesús Angel Ocio Armentia[c], Nico Stollenwerk[a], and Maíra Aguiar †[a,d]

[a]BCAM - Basque Center for Applied Mathematics, Bilbao, Spain
[b]Animal Health Department, NEIKER-Basque Institute for Agricultural Research and Development, Basque Research and Technology Alliance (BRTA), Derio, Bizkaia, Spain
[c]Public Health, Basque Health Department, Rekalde Zumarkalea 39A, 48008 Bilbao, Spain
[d]Ikerbasque, Basque Foundation for Science, Bilbao, Spain

S5 Fig. Scenario-based *Aedes*-borne disease risk maps in the Basque Country, by municipality, for 2019, 2022, and 2023, based on the entomological classification proposed by the Spanish Ministry of Public Health.

All maps were generated using shapefiles from the official resource provided by the Basque Government (Eusko Jaurlaritza / Gobierno Vasco), licensed under Creative Commons CC BY 4.0 `https://www.euskadi.eus/limites-administrativos-del-pais-vasco/web01-ejeduki/es/`.

*bguerrero@bcamath.org
†maguiar@bcamath.org

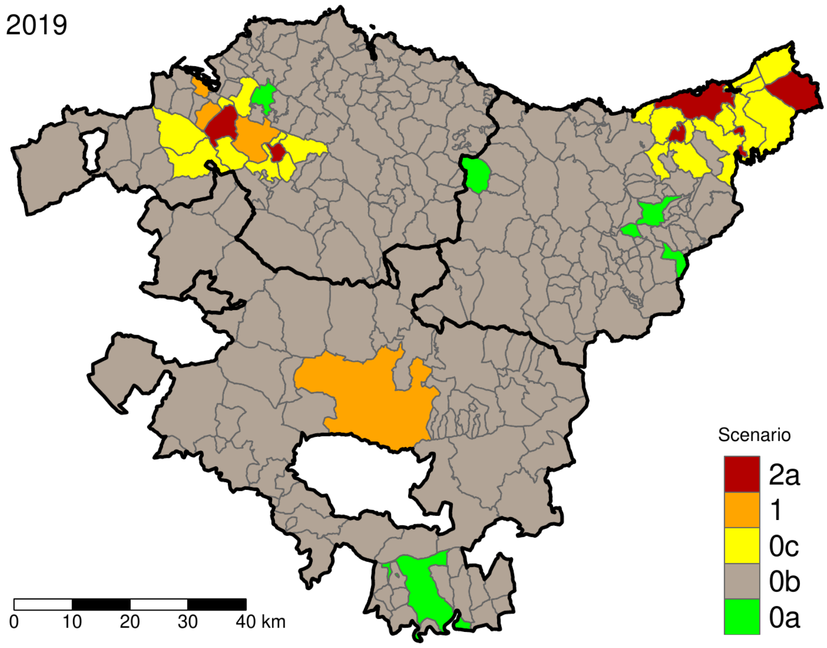
2019
Scenario
2a
1
0c
0b
0a
0
10
20
30
40 km

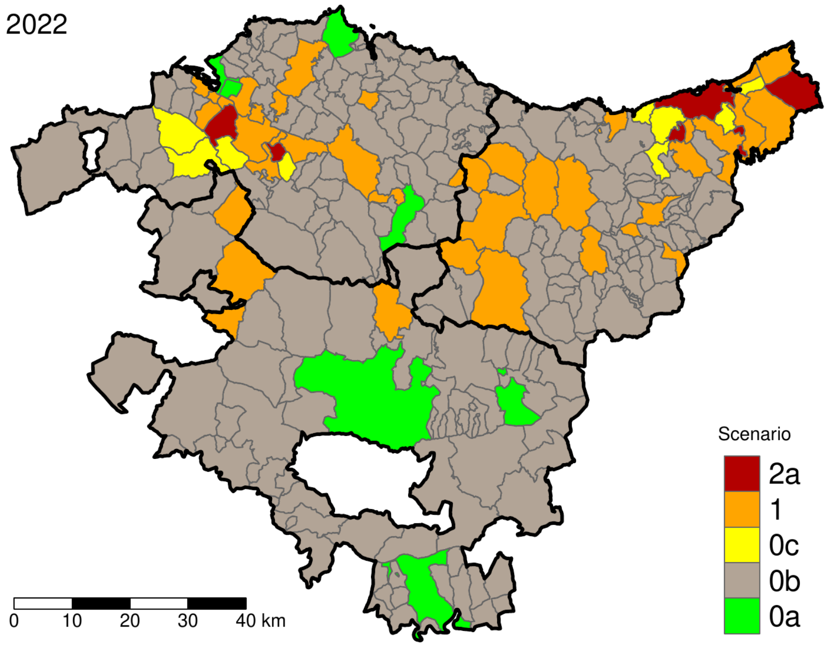
2022
Scenario
2a
1
0c
0b
0a
0
10
20
30
40 km

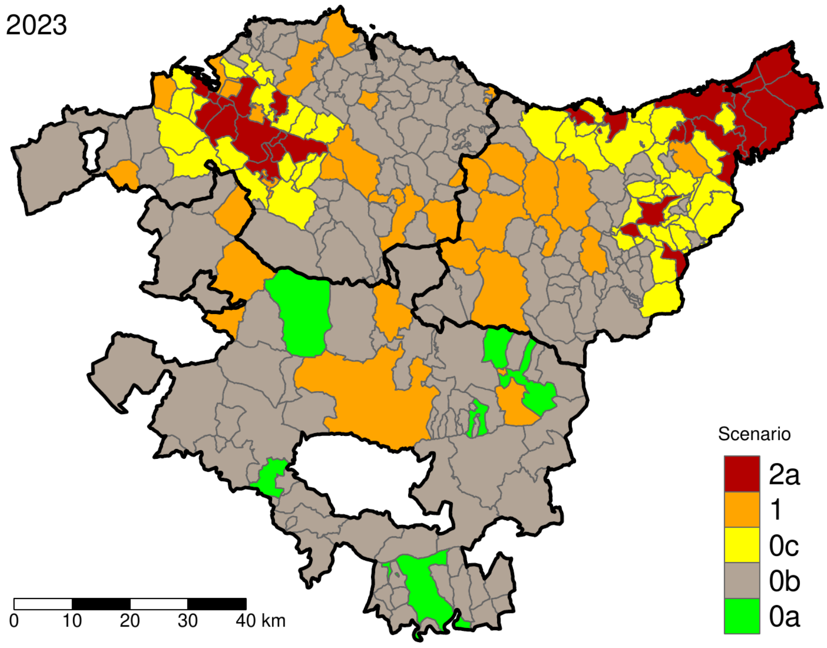
2023
Scenario
2a
1
0c
0b
0a
0
10
20
30
40 km
