# Supplementary material for: Assessing the spatio-temporal risk of Aedes-borne arboviral diseases in non-endemic regions: The case of Northern Spain
Source: PLoS Negl Trop Dis. 2025 Jul 28;19(7):e0013325. doi: 10.1371/journal.pntd.0013325 (PMC12313078; doi:10.1371/journal.pntd.0013325)
Supplement: Attachment — Submitted filename: PlosNTD_arbovirus_report.pdf [file pntd.0013325.s009.pdf]

Comments on the draft “Assessing the Spatio-Temporal Risk of Aedes-Borne Arboviral Diseases in Non- Endemic Regions: The Case of Northern Spain ”by Bruno V. Guerrero, Vanessa Steindorf, Ruben Blasco-Aguado, Luıs Mateus, Aitor Cevidanes, Jesus F. Barandika, Ana Ramirez de La Pecina Perez, Joseba Bidaurrazaga Van-Dierdonck, Jesus Angel Ocio Armentia, Nico Stollenwerk, and Maira Aguiar

PLOS Neglected Tropical Diseases

In this work, the authors conducted a study to investigate the spatio-temporal quantifier to assess the risk of arboviral diseases transmitted by Aedes mosquitoes in non-endemic regions, using the Basque Country as a case study for dengue, Zika, and chikungunya. The risk estimator is derived from the SIRUVY model that incorporates data on mosquito abundance and imported cases, providing an estimate of the expected number of autochthonous cases. The model works under the assumption that non-endemic areas lack local transmission and that the main drivers of outbreaks are imported cases and the vector population. The risk estimator can be easily interpreted that the higher the number of mosquitoes and imported cases, the higher the risk. This approach is simple, relying only on available entomological and epidemiological data. I find the work intriguing and recommend it for publication with some minor revisions. The authors are advised to address the following points before the article’s publication.:

The following is a list of comments:

1. How were the different geographical (municipal vs. provincial) and temporal (monthly vs. annual) scales balanced when synthesizing the risk data? Was the temporal resolution consistent across all municipalities, and how did this affect the generalization process?

2. The author could explain more why did some certain areas, like Bilbao, surpass previous risk peaks, and what implications does this have for future interventions?

3. How can the model be adapted to include the growing trend of urbanization and its effects on mosquito breeding habitats, and what modifications would be necessary to reflect the rapid expansion of cities in non-endemic regions? Can the model incorporate projections of future climate conditions and mosquito population dynamics to provide long-term risk assessments, particularly for regions that are likely to experience increased transmission due to environmental shifts?

4. It would be better to explain the potential consequences of overestimating risk or underestimating risk in the model’s predictions, and how could these errors impact decision-making and resource allocation for vector control?

5. How could the model’s predictions be validated against observed outbreaks in other non-endemic regions, and what steps would be needed to assess its robustness across different geographic settings?

6. Could this risk estimator be integrated into early warning systems for public health authorities, and how might it be combined with other data sources to trigger timely responses?

7. The reviewer is not so clear that if it is possible to explore the effect of incorporating additional epidemiological data such as the movement of populations, human behavior into the risk estimator.

8. Could authors adapt a stochastic modeling approach, rather than the deterministic method cur-

rently used, to better account for the inherent uncertainty in mosquito population dynamics and imported case data?

9. It would be better to explain the gaps in data, such as the lack of mosquito abundance data in colder months if its relevant.

In conclusion, the paper may be accepted for publication after answering these queries.
