# Supplementary material for: Assessing the spatio-temporal risk of Aedes-borne arboviral diseases in non-endemic regions: The case of Northern Spain
Source: PLoS Negl Trop Dis. 2025 Jul 28;19(7):e0013325. doi: 10.1371/journal.pntd.0013325 (PMC12313078; doi:10.1371/journal.pntd.0013325)
Supplement: Attachment — Submitted filename: Rebuttal.pdf [file pntd.0013325.s010.pdf]

Author Response to Reviewers

Assessing the Spatio-Temporal Risk of Aedes-Borne Arboviral Diseases in Non-Endemic Regions: The Case of Northern Spain

Bruno V. Guerrero, Vanessa Steindorf, Rubén Blasco-Aguado, Luís Mateus, Aitor Cevidanes, Jesús F. Barandika, Ana Ramírez de La Peciña Pérez, Joseba Bidaurrazaga Van-Dierdonck, Jesús Angel Ocio Armentia, Nico Stollenwerk, Maíra Aguiar

PLOS Neglected Tropical Diseases, `Manuscript`:**PNTD-D-25-00343**

RC: ***Reviewer Comment***, AR: *Author Response*

We sincerely thank the editor for considering our manuscript and the reviewers for their thorough evaluation and valuable feedback. Their insightful suggestions have greatly contributed to enhancing the clarity and quality of our work. We have carefully addressed all concerns and implemented the corresponding revisions, which we believe have significantly improved the current version paper. Below, we provide detailed responses to each point raised.

We trust that the revised manuscript satisfactorily responds to all reviewer comments and hope that it is now suitable for publication.

Reviewer 1. Comment 1

RC: *Regarding the study data, it is mentioned: "Due to ethical considerations and commercial sensitivity, these data are not publicly available." Given this restriction, is there an Institutional Review Board (IRB) approval or an equivalent ethics committee approval for the use of this data? Clarifying this would help ensure compliance with ethical research standards and data protection guidelines. Additionally, while the data may not be publicly available, it is important to provide a clearer understanding of its nature, scope, and any preprocessing steps taken. This would enhance transparency and allow readers to better assess the study's methodology and findings.*

AR: Thank you for your thoughtful comments regarding data ethics and transparency.

The datasets used in this study were provided by the Basque Country's Public Health Department and NEIKER (Basque Institute for Agricultural Research and Development) under confidentiality agreements. These data are subject to restrictions based on local data protection laws and institutional policies. As such, we are not authorized to publicly share the raw data or include them in the supplementary material.

Regarding ethical approval, the study did not involve identifiable personal data or direct interaction with human or animal subjects. Therefore, Institutional Review Board (IRB) or ethics committee approval was not required under the guidelines of the participating institutions. Nevertheless, all data handling complied with relevant data protection regulations, and the analysis was conducted in line with ethical standards for research using aggregated and anonymized health and surveillance data.

To improve transparency, we have expanded the *Materials and Methods: Geographical scope and data sources* section of the revised manuscript to provide further details about the datasets, including their temporal and geographic scope, and the preprocessing procedures applied before analysis. We believe this information will help readers better understand the study's methodology and interpret the findings, while still honoring the confidentiality constraints imposed by our data providers.

Finally, please note that the **Data Availability Statement** will be updated by the editorial staff based on the information we have supplied. The data may be made available upon reasonable request to researchers who meet the necessary criteria for access to confidential health data and possess appropriate ethics approval.

We appreciate your understanding of these limitations and your attention to ethical research standards.

Reviewer 1. Comment 2

RC: *To enhance the clarity and impact of the study, it is advisable to explicitly state the hypothesis and objectives.*

AR: We thank the reviewer for this valuable suggestion. We agree that the hypothesis and objectives were not sufficiently explicit in the initial version. In this study, we hypothesize that dengue outbreaks in temperate, non-endemic regions can emerge under subcritical vector abundance conditions, driven primarily by the stochastic introduction of imported cases. Our objective is to develop a modeling framework capable of capturing this dynamic - where outbreak occurrence cannot be forecast with certainty, but early warning signals can indicate an increased likelihood of transmission. Strengthening surveillance to detect such signals may prove critical for timely and proactive public health interventions.

In response to the reviewer's comment, we have revised the *Abstract* to explicitly state the study's main hypothesis and objectives. All corresponding modifications are highlighted in blue in the revised version. We trust that these improvements enhance the clarity, coherence, and overall contribution of our study.

Reviewer 1. Comment 3

RC: *It is important to provide general details about the analyzed population, as this contextualizes the study and ensures a better understanding of its scope and applicability.*

AR: We thank the reviewer for this valuable suggestion. In response, we have added clarifying remarks to the *Geographical scope and data sources* section, providing more general details about the analyzed population to better contextualize the study. We believe these additions help address the reviewer's concern and improve the reader's understanding of the study's scope and applicability.

Reviewer 1. Comment 4

RC: *In lines 9-10 when you mention "Our refined SIRUV human-vector compartmental model incorporates stochastic formulations to capture fluctuations in imported cases, mosquito density, and autochthonous transmission risk - critical factors in outbreak emergence" you could include the SIRUVY model as this "Our refined SIRUV human-vector compartmental model (SIRUVY) incorporates stochastic formulations to capture fluctuations in imported cases, mosquito density, and autochthonous transmission risk - critical factors in outbreak emergence" I believe it is important to explicitly state "SIRUVY" in this way, as it highlights the novelty of the model.*

AR: Thank you for the insightful suggestion. We have revised the text to include the model acronym "(SIRUVY)" as recommended, which helps to clearly highlight the novelty of our refined human-vector compartmental model.

Reviewer 1. Comment 5

RC: *The sentence in lines 26 through 28 "Climate change, combined with rising international human mobility and trade, not only facilitates the proliferation of disease vectors but also drives the introduction of pathogens*

into naïve areas, increasing interactions between infected individuals and susceptible populations" could be misinterpreted to suggest that vector-borne diseases (VBDs) are directly transmitted between infected individuals and susceptible populations. To clarify that the transmission occurs through vectors, you might consider rewording it like this: "Climate change, combined with rising international human mobility and trade, not only facilitates the proliferation of disease vectors, but also drives the introduction of pathogens into naïve areas, increasing the risk of local vector-mediated transmission to susceptible populations."

AR: We agree with the reviewer's observation and have revised the sentence to clarify the vector-mediated nature of transmission. We appreciate the helpful suggestion.

Reviewer 1. Comment 6

RC: *In line 33 you could change "arrival of imported cases" for "introduction of imported cases". If you choose to implement this suggestion and decide on a specific term, I recommend using the same term consistently throughout the article, provided this comment is applicable.*

AR: We thank the reviewer for this thoughtful suggestion. After consideration, we have chosen to retain the phrase "arrival of imported cases" as we believe it more accurately conveys the ongoing occurrence of cases over time, whereas "introduction" could be interpreted as referring only to initial cases. We have ensured consistent use of the chosen term throughout the manuscript to maintain clarity.

Reviewer 1. Comment 7

RC: *In line 34 "autochthonous cases—locally transmitted infections" sounds redundant. You could use "locally transmitted infections" instead.*

AR: We appreciate the reviewer's suggestion. We agree that the original wording was somewhat redundant, so we have revised the sentence to keep the definition of autochthonous cases while avoiding repetition.

Reviewer 1. Comment 8

RC: *On lines 75-76, I would use the word adaptively "Once the relevant data are available, this framework can be adaptively applied to other regions experiencing the introduction of Aedes-borne diseases."*

AR: We appreciate the reviewer's insightful suggestion. The sentence has been revised to:

> *"Once the relevant data are available, the framework can be readily adapted to other regions facing the emergence of Aedes-borne diseases."*

Reviewer 1. Comment 9

RC: *On lines 96–97, the description of the database is limited. To enhance clarity and reproducibility, consider including additional details such as the total number of reported cases, the period covered by the dataset, and key variables recorded. Additionally, specify whether the cases are confirmed, suspected, or both. If the dataset is publicly available, please indicate whether it is open-source and provide a reference if possible. Similarly, for the entomological data mentioned on lines 97–98, please clarify the nature of the data provided by NEIKER.*

AR: We thank the reviewer for this helpful and constructive suggestion. In response, we have expanded the *Geographical scope and data sources* section to include further detail on the datasets used in our study. We

now clarify that the human case dataset includes only confirmed cases, and we provide additional information regarding the number of reported cases, the period covered (2019–2023), and key variables recorded.

Similarly, we have elaborated on the entomological data provided by NEIKER, specifying the type of surveillance performed (ovitrap monitoring), the frequency of collection, and the variables included (e.g., egg counts per trap per time unit).

To enhance clarity, **Figure 1** has also been revised to depict both the temporal and spatial distribution of confirmed cases and average *Aedes* egg counts across the study area. In line with the Editorial staff's request, the figure caption now includes a brief note on data availability and reproduction.

As mentioned in our response to *Comment 1*, the raw datasets used in this study are subject to confidentiality agreements and are therefore not publicly available.

Reviewer 1. Comment 10

RC: *On line 106, would it be useful to specify the epidemiological weeks of the years mentioned?*

AR: We thank the reviewer for raising this thoughtful point. While it is indeed possible to map calendar dates to epidemiological weeks, using tools such as the `epical` package in R, there are important considerations that led us to retain calendar-based time references in this study.

First, the definition of epidemiological weeks is not standardized across institutions: for example, the WHO defines weeks as Monday to Sunday, while the CDC uses Sunday to Saturday. Additionally, epidemiological weeks are defined such that the first week of the year must contain at least four days of that year, which means the first and last epidemiological weeks may overlap with adjacent calendar years. This introduces the need to specify both the week number and the corresponding epidemiological year to avoid ambiguity.

Second, although epidemiological weeks are standard in public health, they may not be intuitive for all readers, particularly those from outside the field, such as researchers in mathematics or computer science, or public health managers and administrative authorities. Using standard calendar dates improves readability and accessibility across disciplines, and preserves clarity in communication.

Finally, calendar-based units maintain temporal coherence with existing and future datasets and avoid unnecessary data transformations that do not offer significant interpretive benefits in this context.

For these reasons, we believe that using calendar dates better serves the clarity and interdisciplinary accessibility of our analysis.

Reviewer 1. Comment 11

RC: *In lines 125-133 when you mention "Additionally, there is a steady external import of new infected cases Y, introduced at a constant arrival rate ρ." wouldn't this be deterministic since new cases are introduced at a constant rate? If there is any stochastic variability in the importation process, consider clarifying whether ρ represents an expected average rate at a certain point in time, rather than a strictly fixed value. My understanding is that S0, U0 and Y0, serve as initial conditions that shape the model's behavior. However, when applied in different contexts, they can act as adaptive variables rather than fixed parameters, allowing the model to be used in a contextual rather than a purely replicative way. It is key to emphasize that Y0, is crucial for non-endemic regions where outbreaks are seeded by travelers. The contextual number of imported cases will vary over time depending on travel patterns, seasonality, and other external factors. Based on this, these adaptive variables enable the model to be applied in a contextual, time-based, and adaptive manner*

across different settings, rather than simply replicating it unchanged. It is essential to highlight this point in the article as your audience may not be familiar with this. This would allow to overstress that, as stated in line 63, "deterministic and stochastic models are not designed as direct risk estimators" and as stated in lines 158-159 "However, while this result is derived from a deterministic model, stochasticity is essential to account for the irregular behavior and large fluctuations observed in real disease incidence data"

AR: Thank you for this insightful and constructive comment. You are absolutely right: while the model formulation introduces infected cases at a constant rate ϱ, this parameter is not intended as a strictly deterministic inflow. Instead, it represents the *average* rate of importation over time within a stochastic framework. In our simulations, this aligns with the assumption that imported cases arrive as uncorrelated events, modeled as a *Poisson process*. We have clarified this interpretation in the revised *Determining the expected number of imported cases* $\langle Y \rangle$ section.

Additionally, we agree with your observation regarding the roles of S_0, U_0, and particularly Y_0, which are initially set as starting conditions but serve a broader purpose. In practical applications, these quantities should be interpreted as *adaptive and context-dependent variables*, influenced by factors such as seasonality, travel patterns, and local surveillance data. This is especially critical in *non-endemic settings*, where local outbreaks are typically seeded by *imported cases*.

We have now emphasized this point more clearly in the revised manuscript. Highlighting the contextual and dynamic nature of these parameters supports the broader aim of our framework: not to serve as a static or replicative model, but rather as a *flexible risk assessment tool* adaptable to diverse spatio-temporal scenarios. These clarifications are consistent with our statement in line 63 of the original manuscript, where we note that deterministic and stochastic models are not intended as direct risk estimators, and with lines 158–159, which emphasize the importance of stochasticity in capturing the irregular behavior and large fluctuations observed in real-world disease incidence data.

Reviewer 1. Comment 12

RC: *In lines 173-175 you state "This variability emphasizes the importance of considering stochastic effects in low-endemicity settings, where random fluctuations can greatly determine observed outbreak dynamics and hinder accurate predictions in real-world scenarios." However, in a dynamic system determination would imply that there is an absolute behavior under certain contextual elements. In that regards you could say "This variability emphasizes the importance of considering stochastic effects in low-endemicity settings, where random fluctuations can greatly influence the determinable nature of observed outbreak dynamics and hinder accurate predictions in real-world scenarios."*

AR: We agree that the use of the word "determine" may be misleading in the context of a framework that incorporates both stochastic and deterministic dynamics. To avoid potential misinterpretation, we have revised the sentence accordingly. The updated paragraph also highlights the role of Y_0 in non-endemic settings, as suggested in the previous comment:

> *"This variability emphasizes the importance of accounting for stochastic effects in low-endemicity settings, where random fluctuations can significantly shape the trajectory of individual outbreaks and hinder accurate predictions in real-world scenarios. In non-endemic regions, where outbreaks are expected to be triggered by imported cases, Y_0 plays a critical role - its timing and magnitude can either accelerate or delay the onset of transmission, as illustrated in Fig. 3c."*

We believe this revised wording more accurately conveys the influence of stochastic variability and clarifies

the contextual importance of Y_0 within the model.

Reviewer 1. Comment 13

RC: *In figure 1, the words "imported cases" are overlapping with the maps. You could use some coding to avoid that overlapping.*

AR: Thank you for pointing this out. We have corrected the overlap issue in Figure 1 by adjusting the placement of the labels to ensure better readability. The revised figure and its legend have been updated accordingly in the manuscript.

Reviewer 1. Comment 14

RC: *In line 274: "In 2019 and 2022, the highest egg counts occurred during the warm season - July, August, and September - as anticipated." hot season might be considered too colloquial. A more precise and appropriate term would be "warm season", "summer months", or "peak temperature months".*

AR: Thank you for the suggestion. We have replaced the term with "warm season" to improve clarity.

Reviewer 1. Comment 15

RC: *Regarding Figure 5 and the preceding lines, it is important to consider the broader perspective: climate change is driving environmental shifts that enable mosquitoes to adapt to regions that were previously unsuitable for their survival. While this process is unfolding, and it may be difficult to definitively state that dengue outbreaks are occurring, the changing conditions are increasingly favorable for mosquito populations to thrive. This, in turn, heightens the likelihood of future outbreaks. Although the exact timing of such outbreaks remains unpredictable, the prevailing conditions strongly suggest that their occurrence is becoming more probable. This has significant public health implications, as expanding mosquito habitats may lead to an increased burden of vector-borne diseases in regions that lack prior experience in managing them. Strengthening surveillance, preparedness, and targeted interventions is crucial to mitigating the potential health risks associated with these emerging threats, particularly through a spatiotemporal approach that considers both geographic spread and seasonal variations in risk.*

AR: We agree with the reviewer that this broader perspective is important to include. Accordingly, we have added the following paragraph before the introduction of Figure 5:

> *"Climate change, together with other factors, is altering environmental conditions and enabling mosquitoes to adapt and survive in regions that were previously unsuitable. While disease outbreaks have not yet been confirmed and the timing of potential future outbreaks remains uncertain, these changing conditions increasingly favor mosquito population growth, thereby elevating the risk of outbreaks. This risk is particularly concerning in areas that may be unprepared to face such emerging threats and the associated public health challenges."*

Reviewer 1. Comment 16

RC: *In lines 409-411, you may want to clarify whether the "developing countries" you mention refer to non-endemic countries or those located in the region near Spain. For example, you could revise the sentence as follows: "It is important to note that while not all infected human cases may be properly reported—especially in developing countries, whether non-endemic or those near Spain—this could lead to a slight underestimation*

of the actual number of infections." This clarification ensures that the reader understands the specific context of underreporting.

AR: We appreciate the reviewer's helpful suggestion. In the original text, the term "developing countries" was used in a broad, global sense and was not intended to refer specifically to countries near Spain. To avoid any potential confusion, we have revised the paragraph to clarify the context of underreporting and have removed the term "developing countries" from the revised manuscript.

Reviewer 1. Comment 17

RC: *While the current title is clear and informative, incorporating key elements from the article could further enhance its specificity and impact. A suggested title revision is: "Assessing the Spatio-Temporal Risk of Aedes-Borne Arboviral Diseases in Non-Endemic Regions: A Data-Driven SIRUVY Model Approach in Northern Spain." This revised title highlights both the methodological approach (SIRUVY model) and the geographic focus, reinforcing the study's innovative contributions.*

AR: We thank the reviewer for the insightful suggestion regarding the title. However, we consider the current title to be sufficiently clear and informative, and have therefore decided to retain it unchanged. Including extensive model acronyms in the title may reduce clarity and accessibility for a broad readership.

NB: Please note that in the "Summary and General Comments" section of the reviewers' report, Reviewer 1's Comments 4–12 were duplicated. We have not repeated them here, as these points have already been addressed in our responses to Reviewer 1 in this rebuttal letter.

Reviewer 1. Comment 18

RC: *While the article discusses imported cases and meteorological conditions, it does not consider other factors that contribute to mosquito proliferation. This is crucial to consider, as these additional factors add to the complexity of the situation and may help in formulating hypotheses or providing a more comprehensive explanation of the dynamics at play. Key factors that should also be taken into account include land use and urbanization patterns, water storage and management practices and socio-economic conditions. These elements can significantly influence mosquito breeding, survival, and disease transmission, making them essential for a more holistic risk assessment.*

AR: We appreciate the reviewer's insightful comment. In fact, the manuscript already acknowledges the multi-factorial nature of mosquito proliferation beyond meteorological conditions. Specifically, the *Introduction* highlights that mosquito-borne diseases are influenced by a variety of environmental and socio-demographic factors, including land use, urbanization, water storage practices, human activity, and biological interactions:

> " Mosquito-borne diseases pose a significant global health threat, exacerbated by climate change and global warming. The transmission of these diseases, mediated by mosquitoes, is highly sensitive to climatic factors that affect both vector adaptability and pathogen development [1,2,3,4,5]. Environmental changes and socio-demographic shifts, such as deforestation and urbanization, are further contributing to the expansion of both emerging and resurgent diseases [3,6,7]."

Our SIRUVY-based risk model focuses primarily on mosquito abundance and the mean number of imported infected cases. This parsimonious approach is especially valuable in data-scarce contexts where detailed information on additional covariates may be unavailable, uncertain, or lacking altogether. By relying on

empirical records of mosquito abundance and imported infections, the model implicitly captures the combined effects of various environmental, ecological, and biological factors - such as temperature, humidity, host availability, land use, demographic changes, and vector control measures - without explicitly modeling each individually. This strategy enhances the model's realism and robustness while maintaining analytical simplicity and reducing potential errors linked to covariate mis-specification.

We acknowledge that when more comprehensive and longitudinal data on relevant covariates become available, future model extensions could explicitly incorporate these variables - such as seasonality effects through periodic transmission rates or vector capacity functions dependent on temperature, rainfall, and habitat suitability. However, given the absence of mosquito presence during colder months in our Basque Country case study, the current simplified framework remains appropriate and effective for capturing key transmission dynamics.

These considerations have been incorporated into both the *Introduction* and *Discussion* sections of the revised manuscript to provide a clearer context for our modeling approach.

Reviewer 1. Comment 19

RC: *It is important to document the analysis software and the specific packages used in the study. Please provide references for them to ensure transparency, reproducibility, and clarity for readers who may want to replicate or build upon the analysis.*
While the data source is mentioned, it is important to document how the data was processed and prepared for analysis. Providing details on these steps ensures transparency, reproducibility, and a better understanding of the methodological approach used in the study.

AR: We thank the referee for emphasizing the importance of transparent documentation of the analysis software and data processing steps to ensure reproducibility.

All parameter values used in the numerical simulations and risk estimations are comprehensively listed in Table 1 and described in the main text.

Regarding geographic data, the administrative boundary maps for the Basque Autonomous Community were obtained from the official Basque Government resource (Eusko Jaurlaritza / Gobierno Vasco), provided under a Creative Commons CC BY 4.0 license. These shapefiles are referenced to the ETRS89 datum and projected in UTM Zone 30, covering municipalities, counties, functional areas, historical territories, and the autonomous community boundaries. The source is cited in the revised manuscript as reference [44] [Government of the Basque Country2025], which links to:

`https://www.euskadi.eus/limites-administrativos-del-pais-vasco/web01-ejeduki/es/`.

All data processing, analysis, and visualization were performed using R, relying exclusively on base R functions and widely used, well-documented packages from the official repositories. No custom or proprietary code was used; all tools and routines are standard in mathematical epidemiology and spatial analysis.

The datasets were prepared as plain CSV files containing key fields such as case ID, disease type, date, location (latitude and longitude), ovitrap ID, egg counts, mosquito species, and relevant spatial-temporal metadata, as described in the manuscript's *Geographical scope and data sources* section. Data cleaning involved standard wrangling steps to organize and format these files for analysis.

Due to confidentiality agreements with data providers, raw datasets cannot be publicly shared. Nonetheless,

our methodology is fully reproducible: the deterministic (ODE-based) and stochastic (Gillespie algorithm) simulation approaches are well-established, and the mapping procedures follow standard practices available across common platforms such as R, Python, MATLAB, QGIS, ArcGIS, and Tableau.

To enhance transparency, the revised manuscript now includes a dedicated section, *On spatio-temporal scales for risk assessment*, detailing the rationale behind spatial and temporal resolution choices, data aggregation methods, and processing workflows.

Given the wide availability of theoretical and computational tools used, the choice of software does not constrain reproducibility. Researchers with their own data can readily apply our framework using any standard computational environment.

We hope these clarifications address the referee's concerns while maintaining compliance with data use agreements and ethical standards.

Reviewer 1. Comment 20

RC: *While reviewing the article, it appears that the complexity of the situation is addressed through elements of either a systems approach or complexity theory. This is a valuable perspective; however, it would be ideal to emphasize this more explicitly, particularly in relation to complex adaptive systems and emergent phenomena. Highlighting this framework could enhance the understanding of the situation and support the development of future actions based on a more nuanced and dynamic vision, where agents interact in unpredictable ways. However, the key aspect to consider is not predictability itself, but rather how environmental changes facilitate mosquito adaptation. In this context, dengue outbreaks should be understood as emergent phenomena, meaning their occurrence can be anticipated not through precise forecasting, but by identifying early signals that indicate the increasing likelihood of such events. Strengthening surveillance efforts to detect these signals could be instrumental in proactive public health interventions.*

AR: We thank the reviewer for this thoughtful and constructive comment. We fully agree that **dengue outbreaks in non-endemic regions** can be understood as emergent phenomena within complex adaptive systems, where dynamic interactions between agents, such as human hosts, vectors, and environmental conditions, give rise to nonlinear and often unpredictable patterns. In this context, **the key challenge is not to forecast the exact timing of an outbreak, but rather to detect early signals that indicate an increasing likelihood of such events, thereby enabling proactive public health responses**.

As illustrated in Figure 3, even when initial conditions are well characterized, the inherent stochasticity of the system makes precise prediction of outbreak timing in individual realizations fundamentally difficult. Our proposed risk estimator directly addresses this challenge by providing a probabilistic assessment of outbreak likelihood, serving precisely as the type of early warning signal highlighted by the reviewer. Higher risk estimates indicate a greater likelihood of outbreak occurrence and can inform timely and targeted interventions.

In the original manuscript, we explicitly articulated this conceptual framework. In the revised version of the paper, we have further elaborated on these ideas. In the *Introduction*, we present a brief overview of the various methodological paradigms used in public health risk assessment, including systems thinking and complexity-informed approaches. In the *Conclusions* section, we expand on how our proposed tool integrates into a broader surveillance ecosystem, emphasizing its strengths as a simple, interpretable, and data-driven method—particularly well-suited for data-scarce, non-endemic settings.

We underscore that our framework is not intended to replace more comprehensive or mechanistic models,

but to complement them, especially when combined with state-of-the-art hybrid strategies for vector-borne disease surveillance. By facilitating the identification of early outbreak signals, our approach can enhance the responsiveness of public health systems and support more effective, anticipatory interventions.

We appreciate the opportunity to clarify these important points and have ensured that they are more explicitly presented in the revised manuscript.

Reviewer 3. Comment 1

RC: *Dr. Guerrero and colleagues have presented an interesting article addressing the epidemiological risk of Aedes-borne arboviral diseases in Northern Spain. While the mathematical modeling is theoretically robust, there are significant concerns regarding the assumptions underlying the model, which, in their current form, compromise the reliability and practical utility of the risk estimates. These assumptions should be thoroughly revised or any statement on the usefulness for risk estimation and applicability considerably tuned down.*

AR: We thank the referee for raising important concerns about the assumptions underlying our model and their potential impact on the reliability and practical relevance of the risk estimates. In the responses to the specific points below, we provide detailed justifications for the assumptions adopted - many of which were deliberately chosen to be conservative. **We also demonstrate that these assumptions do not undermine the robustness or applicability of our risk assessment approach for non-endemic settings**, as the methodology remains flexible and informative under a range of plausible scenarios.

RC: *In particular:*

1. The model assumes a constant external importation rate of infected individuals. This assumption is likely unrealistic, as importation rates are expected to vary seasonally, reflecting epidemic dynamics in endemic regions and fluctuations in tourist travel patterns. Furthermore, imported cases may be spatially clustered, particularly in high-density or high-income areas. A time-dependent importation rate might show that most cases arrive during periods when transmission conditions in Northern Spain are unsuitable, or in areas less suitable to disease spread—potentially lowering the actual risk. Notably, the study does not provide any figures or data illustrating the number or distribution of imported cases.

AR: We appreciate the referee's thoughtful observations and the opportunity to elaborate on this important modeling assumption. While we understand the concerns raised, we respectfully offer a different perspective on the use of a constant importation rate:

- *"The model assumes a constant external importation rate of infected individuals. This assumption is likely unrealistic..."*

  In our framework, the importation process is modeled as a homogeneous Poisson process, which provides a tractable first-order approximation based on the assumptions of event independence and exponentially distributed inter-arrival times. This simplification is commonly used in stochastic epidemic modeling—particularly when the available data are sparse and lack sufficient temporal granularity (see [Stollenwerk2025]).

  While we acknowledge that importation events may indeed vary seasonally or spatially, the empirical data available (see Fig. 1 and Fig. 3a) typically include no more than 6 events per month. This low incidence limits the statistical power of formal goodness-of-fit tests (e.g., Kolmogorov–Smirnov for inter-arrival times or Chi-square tests for counts). As such, we consider the constant rate assumption to be a conservative, pragmatic choice in the absence of strong evidence supporting more complex time-dependent or spatially structured alternatives.

  We agree that more granular temporal or spatial importation data, if available, could improve the realism of the model, and we are open to incorporating such refinements in future work. For now, we believe that the assumption of a constant importation rate does not significantly compromise the validity or usefulness of the proposed risk assessment approach in non-endemic settings. Please see the highlighted section *Determining the expected number of imported cases* $\langle \mathbf{Y} \rangle$ for further clarification.

- ... *"as importation rates are expected to vary seasonally, reflecting epidemic dynamics in endemic regions and fluctuations in tourist travel patterns. Furthermore, imported cases may be spatially clustered, particularly in high-density or high-income areas. A time-dependent importation rate might show that most cases arrive during periods when transmission conditions in Northern Spain are unsuitable, or in areas less suitable to disease spread—potentially lowering the actual risk."*

AR: We acknowledge that importation rates in non-endemic settings are expected to show some degree of seasonal variation, driven by epidemic activity in endemic regions and international travel dynamics. However, **in our context, the number of imported cases observed within each time interval is low, which limits our ability to detect or robustly confirm seasonal or spatial trends**. As shown in Fig. 1a, while some fluctuation is visible in the monthly number of reported imported cases, particularly the drop during the COVID-19 lockdown (light purple-highlighted period), no clear seasonality emerges that would justify introducing a more complex time-varying importation structure.

In addition, **the actual number of imported infections is likely underestimated due to asymptomatic or mildly symptomatic cases**, which frequently go undetected, especially in regions where arboviral diseases are not yet routinely screened for. This underreporting may further obscure any underlying seasonal or spatial patterns and is a known challenge in arboviral surveillance (see *Discussion*).

While non-Poissonian models can capture richer importation dynamics, they require stronger and more specific assumptions about the underlying temporal trends—assumptions that cannot be supported here due to the sparse nature of the data. Importantly, imported cases into Northern Spain originate from multiple endemic regions located in both hemispheres. This geographic diversity results in partially overlapping epidemic seasons, which can blur clear seasonal signals in the aggregated importation data. Moreover, even during periods with expected higher global transmission activity, few or no cases are often recorded in our study setting.

These factors, including sparse observations, potential underreporting, and overlapping transmission dynamics, complicate efforts to reliably model seasonal importation patterns and support our decision to use a constant-rate Poisson process as a conservative, data-informed simplification.

As previously outlined in the original manuscript:

> "In a non-endemic setting, the random arrival of viremic imported cases can be modeled as a Poisson process, under the premise that these events occur independently at a constant effective rate λ."

This assumption is further supported by the explanations now included in the revised manuscript. By acknowledging the expected seasonal variability and clearly articulating the limitations posed by narrow time windows and sparse data, we strengthen the justification for adopting a simplified Poisson process framework. This modeling choice reflects both the practical constraints of the available data and the low number of observed cases, which hinder the reliable estimation of more complex time-varying importation structures. The revised discussion also emphasizes that these limitations are common in non-endemic regions and highlights the value of conservative assumptions in generating robust and interpretable risk estimates under such conditions.

- *"Notably, the study does not provide any figures or data illustrating the number or distribution of imported cases."*

We respectfully disagree with this statement, as the original manuscript does include figures illustrating both the number and distribution of imported cases. Specifically:

- Figure 1a shows the monthly time distribution of imported cases aggregated at the provincial level.
- Figure 1b and the maps in the *Supplementary Material* present the monthly spatial distribution of imported cases at the municipal scale.

These figures provide a clear overview of both temporal and geographic patterns of importation.

Additionally, Figure 3a presents a synthetic time series that mirrors the typical behavior of imported cases observed at the municipal level, where the number of cases per month is typically low (up to six cases, as shown in the maps). The remaining subfigures in Figure 3 illustrate how time series might appear under alternative non-endemic scenarios with varying mosquito abundance, offering readers a broader understanding of expected importation dynamics.

We hope this clarification addresses the concern, and we respectfully note that the imported case data are presented.

RC: *2. The estimation of adult mosquito based on ovitrap data is questionable (see recent review on the AJTMH https://doi.org/10.4269/ajtmh.20-0781 "Its data indicate that it is suitable for assessing the presence of Ae. albopictus at a given site, but not adult abundance") and raises concerns. It seems to me that, in the end, authors relied on an arbitrary value of kend = 1000 (or its half). Additionally, despite it being a key point in their model, only the three highest positive mosquito egg counts per oviposition stick are reported in the Results section. This limited dataset is inadequate to characterize the mosquito population in Northern Spain or to draw meaningful conclusions about epidemiological risk.*

AR: We thank the referee for the opportunity to clarify both the rationale for using ovitrap data and the scope of our dataset.

Regarding the estimation of adult mosquito abundance from ovitrap data, we acknowledge the referee's concern and the findings of the Liu et al. AJTMH review [Liu2023]. As correctly noted, the authors emphasize that ovitraps are more suited for assessing the presence of *Aedes albopictus* rather than its absolute adult abundance. This interpretation is consistent with our own.

However, the same review also underscores the practical relevance of ovitraps in operational contexts:

> *"the number of eggs in OT is usually regarded as the only indicator of high nuisance or high risk of disease transmission and is used to plan mosquito control operations."*

This reflects the widespread use of ovitrap data in public health programs, particularly in non-endemic regions or early invasion settings, where ovitraps offer high sensitivity, minimal maintenance, and low operational costs. As noted in the revised manuscript, we now cite [Liu2023] explicitly and include this perspective in the section *Determining the mosquito abundance* κ.

From a modeling perspective, it is important to clarify that the vector abundance parameter κ (Eq. 3) is dimensionless and does not depend on the specific sampling protocol. This is a feature of the model that allows consistent application across different surveillance schemes, as long as internal consistency is maintained.

We also acknowledge the referee's concern regarding the reference value $\kappa_{\text{end}} = 1000$. This value is introduced explicitly as a scaling parameter, not as an empirical estimate. As stated in the original manuscript:

"For simplicity, we assume $\kappa_{\text{end}} = 1000$, noting that alternative values would act merely as scaling factors when computing $\langle I \rangle$ and do not affect relative comparisons. In this study, to preliminarily account for annual seasonality, we adjust κ_{20} by a factor of $\frac{1}{2}$."

To illustrate: if one instead uses $\kappa_{\text{end}} = 3000$, this would simply rescale the absolute risk values by a factor of one-third. Crucially, relative risk comparisons across space and time remain unaffected. These relative differences are what guide public health prioritization and early warning strategies.

Regarding the reported data, we regret any confusion. The three highest egg counts per ovitrap were shown in the *Results* section as representative peak activity values. However, these are not the only data used in the analysis. As detailed in:

- Figures 1b–1d (time series and geographical distributions),
- the *Supplementary Material* (detailed municipal maps), and
- the averaged metrics across sites,

our study is based on a high-resolution dataset covering multiple years and municipalities. In each sampling location, five ovitraps were deployed and monitored biweekly, providing a dense spatial and temporal record of *Aedes* mosquitoes activity across the Basque Country.

We thus clarify that the full dataset underpins our analysis, and the metrics reported in the manuscript reflect aggregated measures derived from these broader data.

In conclusion, we agree that ovitraps have limitations for estimating absolute adult abundance. However, their practical utility in non-endemic areas - as early warning tools and as proxies for mosquito activity - is well supported by the literature and operational practices. Our model is constructed to be robust to these limitations, focusing on internally consistent, relative comparisons of risk. The reference value κ_{end} acts only as a scaling parameter and does not compromise the validity of the results. We have revised the manuscript to make these points clearer and trust this response addresses the referee's concerns.

RC: *3. The parameter values used in the model (as listed in Table 1) are not supported by any cited scientific literature. The absence of justification or references undermines the credibility and reproducibility of the model's outcomes.*

AR: We appreciate the referee's concern regarding the justification of parameter values. We respectfully note that two scientific publications supporting these values are already cited in the caption of Table 1 in the manuscript.

The reasoning behind the specific parameter choices is as follows:

- The value $\hat{\varrho} \sim 10^{-5}$ reflects the ratio between the typical monthly number of imported cases (a few units) and the total population of the Basque Country (approximately 10^6). This rationale is now explicitly stated in the revised Table 1 caption.
- Parameters marked with an asterisk were used in the numerical simulations and are well justified within the context of a non-endemic area. For example, $S_0 = 0$ assumes a naïve population, consistent with the Basque Country currently being considered unexposed to local transmission. The number of imported cases (Y) may be zero at a given time and location, as evidenced by the corresponding maps.

- The initial mosquito population $U_0 = mN$ was set based on the scaling factor m, which was varied in simulations to capture the observed dynamics. The sensitivity of this parameter and further mathematical details are thoroughly discussed in our previous study [Stollenwerk2025], which is cited in the manuscript.
- The only true free parameter is κ_{end}, a proportionality constant used to scale absolute risk values. As explained in our earlier response, it does not influence the relative comparison of risk and therefore does not compromise the credibility or reproducibility of the results.

To improve clarity, we have revised the manuscript to better reflect these justifications and explicitly reference them in the relevant table. Although not all parameter assumptions are explicitly detailed in the main text, they are contextually supported and grounded in the epidemiological characteristics of non-endemic regions. For instance, the assumptions of a naïve population or zero imported cases at specific times align with the epidemiological situation in the Basque Country and are reflected in the maps and model outputs.

While the precise value of the proportionality constant may vary depending on mosquito surveillance protocols used in endemic regions, such variation affects only the absolute scale of risk - not the relative spatio-temporal patterns, which remain robust. This relative risk estimation is a key strength of our approach and underpins its relevance for public health planning and early warning systems.

(shared as PDF)

Reviewer 4. Comment 1

RC: *In this work, the authors conducted a study to investigate the spatio-temporal quantifier to assess the risk of arboviral diseases transmitted by Aedes mosquitoes in non-endemic regions, using the Basque Country as a case study for dengue, Zika, and chikungunya. The risk estimator is derived from the SIRUVY model that incorporates data on mosquito abundance and imported cases, providing an estimate of the expected number of autochthonous cases. The model works under the assumption that non-endemic areas lack local transmission and that the main drivers of outbreaks are imported cases and the vector population. The risk estimator can be easily interpreted that the higher the number of mosquitoes and imported cases, the higher the risk. This approach is simple, relying only on available entomological and epidemiological data. I find the work intriguing and recommend it for publication with some minor revisions. The authors are advised to address the following points before the article's publication:*

AR: We thank the referee for the constructive summary and positive evaluation of our work. We appreciate the recommendation for publication and the thoughtful suggestions provided. While some of the points raised extend beyond the intended scope of this study, they nonetheless help to contextualize our findings within the broader field of arboviral risk assessment. We have carefully addressed all comments and revised the manuscript accordingly to improve clarity and robustness.

Reviewer 4. Comment 2

RC: *How were the different geographical (municipal vs. provincial) and temporal (monthly vs. annual) scales balanced when synthesizing the risk data? Was the temporal resolution consistent across all municipalities, and how did this affect the generalization process?*

AR: Thank you for your insightful question. We believe the following paragraph addresses your concerns and has been included in the *Conclusions* section of the revised manuscript to explicitly acknowledge and account for these limitations.

> *"Applied to the Basque Country and tailored to the available data, our framework accommodates different spatial (municipal, provincial) and temporal (monthly, annual) scales, producing time series and choropleth maps to guide targeted public health interventions."*

Reviewer 4. Comment 3

RC: *The author could explain more why did some certain areas, like Bilbao, surpass previous risk peaks, and what implications does this have for future interventions?*

AR: We appreciate this thoughtful question. A concise explanation has been added to the *Conclusions* section of the revised manuscript:

> *"Our findings, based on entomological and epidemiological data up to 2023, reveal an upward trend in both mosquito abundance and imported viremic cases. Risk estimates highlight Irun and neighboring municipalities as persistent hotspots since 2013, with recent increases in vector presence shifting the highest risk levels to Bizkaia. Despite a doubling of risk levels from 2022 to 2023, no local cases have been reported to date, and mosquito activity remains absent in colder*

months, reaffirming the Basque Country's non-endemic status. These observed trends highlight the limitations of linear forecasting methods and underscore the importance of continuous, data-driven risk assessments to capture the evolving patterns of vector spread and associated disease risks."

Additionally, beyond the climatic and environmental factors mentioned in the *Introduction*, [Cevidanes2023] examined the progression of *Aedes* invasion in Euskadi in relation to urbanization patterns and the coexistence of *Aedes albopictus* and *Aedes japonicus* populations. While these aspects are acknowledged in response to the reviewer's interest, we refrain from further speculation on entomological mechanisms, as such analysis lies beyond the scope of the present study and could detract from its focus and methodological integrity.

Reviewer 4. Comment 4

RC: *How can the model be adapted to include the growing trend of urbanization and its effects on mosquito breeding habitats, and what modifications would be necessary to reflect the rapid expansion of cities in non-endemic regions? Can the model incorporate projections of future climate conditions and mosquito population dynamics to provide long-term risk assessments, particularly for regions that are likely to experience increased transmission due to environmental shifts?*

AR: We thank the reviewer for this forward-looking question. While a detailed exploration of urbanization trends and climate projections lies beyond the scope of the present study, we acknowledge the importance of these factors in shaping future mosquito-borne disease risk.

To this end, the *Discussions* section includes a curated list of recent modeling approaches that incorporate environmental covariates and human mobility patterns for long-term risk assessment.

It is worth noting that our framework could be adapted for forecasting by integrating projected mosquito abundance and imported case scenarios. However, as noted earlier, the rapid year-to-year variation in mosquito presence and distribution indicates that linear extrapolations may not be reliable, particularly for medium- to long-term projections.

To emphasize the relevance of sustained surveillance under changing environmental conditions, the following paragraph has been added to the *Discussions* section:

"Continuous entomological surveillance is essential in regions undergoing environmental or urban change, as exemplified by the Basque Country's monitoring efforts since the detection of Aedes albopictus in 2013. The adaptability of our framework can be further enhanced by integrating environmental covariates affecting mosquito suitability [30, 59, 60, 53, 61, 62, 63, 64, 38], human mobility patterns and traveler influx [65, 66, 9, 67], as well as advanced spatiotemporal modeling techniques. Hybrid and multimodel approaches, supported by increasingly harmonized multisource datasets [68], would improve the reliability of risk assessments, strengthen surveillance, and refine scenario forecasting, further enhancing public health planning within the conceptual framework developed here."

We hope this clarifies the adaptability of our model framework and its relevance in a changing epidemiological landscape.

Reviewer 4. Comment 5

RC: *It would be better to explain the potential consequences of overestimating risk or underestimating risk in the model's predictions, and how could these errors impact decision-making and resource allocation for vector control?*

AR: We appreciate the reviewer's suggestion. While the aim of this study is not to provide exact risk predictions, but rather to propose a practical tool for risk assessment in non-endemic regions, we recognize the importance of acknowledging the implications of its use in public health planning. To that end, we have added the following paragraph to the *Discussions* section of the revised manuscript:

> "As a comparative and data-informed framework, our tool is intended to support the prioritization of vector surveillance and early intervention, rather than to provide precise forecasts of outbreak magnitude. While any risk assessment system may be sensitive to uncertainties in input data or assumptions, our model emphasizes relative changes and spatial trends rather than absolute case counts. This design helps reduce the risk of misinterpretation and supports its use as a complementary input for public health decision-making, especially in non-endemic settings where timely and targeted responses are critical."

We trust this clarifies the intended scope and practical application of the proposed model.

Reviewer 4. Comment 6

RC: *How could the model's predictions be validated against observed outbreaks in other non-endemic regions, and what steps would be needed to assess its robustness across different geographic settings?*

AR: To validate the model and assess its robustness across non-endemic regions, methodological consistency is essential. This includes standardized mosquito surveillance protocols (e.g., ovitrap data with comparable identification procedures), consistent spatio-temporal scales, and the use of a common or rescaled value for the parameter $kappa_{end}$. Equally important is the availability of epidemiological data on imported cases at compatible spatial and temporal resolutions. However, such harmonized and high-resolution data remain limited in many non-endemic settings, posing a significant challenge.

Broader application of the framework would also require adjusting initial conditions - such as population size and the presence of autochthonous cases - to fit the local context. While the model is flexible, its predictive reliability depends heavily on the quality and availability of input data.

To reflect these points, we have added the following paragraph to the *Conclusions* section:

> *"Broader application of this framework requires standardized, high-resolution entomological and epidemiological data, which are often lacking in many regions. Beyond its methodological value, this work emphasizes the need to strengthen surveillance systems. Improved data availability will not only enhance local risk assessments but also support cross-regional analyses and timely public health responses amid increasing environmental and urbanization pressures."*

Reviewer 4. Comment 7

RC: *Could this risk estimator be integrated into early warning systems for public health authorities, and how might it be combined with other data sources to trigger timely responses?*

AR: The most straightforward way to integrate this risk assessment methodology into early warning systems is via interactive dashboards. These platforms can display multiple layers, including maps and time series of customizable variables, which can be overlaid with alternative scenario-based risk maps, climatic, environmental, geographical, and urbanization data. Such visualization tools facilitate comprehensive monitoring and interpretation, enabling public health authorities to identify emerging risks and trigger timely, informed responses.

Reviewer 4. Comment 8

RC: *The reviewer is not so clear that if it is possible to explore the effect of incorporating additional epidemiological data such as the movement of populations, human behavior into the risk estimator.*

AR: Incorporating such additional data would require either extending the model equations to explicitly include these factors or using them as proxies to estimate mosquito abundance or imported cases, provided that the functional relationships between these variables and the model parameters are understood. We consider this an important direction for future work to improve the model's predictive capabilities.

Reviewer 4. Comment 9

RC: *Could authors adapt a stochastic modeling approach, rather than the deterministic method currently used, to better account for the inherent uncertainty in mosquito population dynamics and imported case data?*

AR: The numerical simulations presented in Fig. 3 are based on a stochastic modeling framework using the Gillespie algorithm. The full mathematical formulation, including the link between stochastic and deterministic models and the associated likelihood calculations, is detailed in a recent paper by us [Stollenwerk2025], which was initially available on arXiv and has now been published. For clarity and ease of interpretation in public health contexts, the deterministic model and its derived risk formula are emphasized in this work.

Reviewer 4. Comment 10

RC: *It would be better to explain the gaps in data, such as the lack of mosquito abundance data in colder months if its relevant. In conclusion, the paper may be accepted for publication after answering these queries.*

AR: We thank the reviewer for this important suggestion. In response, we have added a brief explanation in the *Geographical Scope and Data Sources* subsection to address these data gaps:

> *"During colder months (December to May), mosquito presence was not detected, as confirmed by year-round monitoring at four sentinel sites (two in Gipuzkoa and two in Bizkaia), which were established to track the seasonal activity of Aedes mosquitoes in previously affected regions. "*

We sincerely thank the reviewers and the editor for their valuable feedback and constructive suggestions. Having thoroughly addressed all comments and incorporated the relevant revisions, we believe the manuscript now fulfills the journal's publication criteria.
